# Therapeutic Effects of Vitamin D on Vaginal, Sexual, and Urological Functions in Postmenopausal Women

**DOI:** 10.3390/nu15173804

**Published:** 2023-08-30

**Authors:** Mohammed M. Hassanein, Hasniza Zaman Huri, Abduelmula R. Abduelkarem, Kauser Baig

**Affiliations:** 1 Department of Clinical Pharmacy and Pharmacy Practice, Faculty of Pharmacy, Universiti Malaya, Kuala Lumpur 50603, Malaysia; s2163718@siswa.um.edu.my; 2 Department of Pharmacy Practice and Pharmacotherapeutics, College of Pharmacy, University of Sharjah, University City Road—University City, Sharjah P.O. Box 27272, United Arab Emirates; aabdelkarim@sharjah.ac.ae; 3 Research Institute for Medical and Health Sciences, University of Sharjah, University City Road—University City, Sharjah P.O. Box 27272, United Arab Emirates; 4 Department of Obstetrics and Gynecology, University Hospital Sharjah, Sharjah P.O. Box 27272, United Arab Emirates; kauser.baig@uhs.ae

**Keywords:** menopause, sexual health, therapeutic use, urinary tract infections, vitamin D, vitamin D deficiency, vitamin D receptor, vagina

## Abstract

Recent years have witnessed the emergence of growing evidence concerning vitamin D’s potential role in women’s health, specifically in postmenopausal women. This evidence also includes its connection to various genitourinary disorders and symptoms. Numerous clinical studies have observed improvements in vulvovaginal symptoms linked to the genitourinary syndrome of menopause (GSM) with vitamin D supplementation. These studies have reported positive effects on various aspects, such as vaginal pH, dryness, sexual functioning, reduced libido, and decreased urinary tract infections. Many mechanisms underlying these pharmacological effects have since been proposed. Vitamin D receptors (VDRs) have been identified as a major contributor to its effects. It is now well known that VDRs are expressed in the superficial layers of the urogenital organs. Additionally, vitamin D plays a crucial role in supporting immune function and modulating the body’s defense mechanisms. However, the characterization of these effects requires more investigation. Reviewing existing evidence regarding vitamin D’s impact on postmenopausal women’s vaginal, sexual, and urological health is the purpose of this article. As research in this area continues, there is a potential for vitamin D to support women’s urogenital and sexual health during the menopausal transition and postmenopausal periods.

## 1. Background

Menopause is characterized by many physiological and cellular changes in the external genitalia and urogenital tissues, including the vaginal epithelium, pelvic floor muscles, and urinary tract. These changes are clinically manifested by a broad spectrum of vaginal, urological, and sexual signs and symptoms that are mainly secondary to the hypoestrogenic state after menopause [1,2]. Changes in the vaginal flora, including a decrease in beneficial *lactobacillus* bacteria, lead to an increase in vaginal pH. These changes can cause vaginal dryness, itching, and irritation. The increase in the vaginal pH, in addition to changes in the innate defenses, promotes the overgrowth of harmful bacteria, potentially resulting in urinary tract and vaginal infections. Moreover, loss of the dermal collagen in the connective tissues of the vagina, bladder, and urethra leads to dyspareunia and other urinary symptoms such as dysuria, frequency, and urgency [1,3,4,5,6]. Collectively, these symptoms and signs are known as the genitourinary syndrome of menopause (GSM) [7]. GSM, although highly prevalent as reported by many international surveys [8,9,10,11,12,13], women frequently report symptoms due to sexual embarrassment and thus go untreated [14].

There is a growing focus on exploring the potential of vitamin D as a supplementary approach to hormonal therapies for managing symptoms associated with GSM. This perspective gains support from the observed links between vitamin D deficiency and various complications experienced by postmenopausal women, such as vaginal atrophy [15], sexual dysfunction [16,17], and urogenital infections [18]. Mediated by the vitamin D receptor (VDR), the effects of vitamin D are exerted locally within reproductive tissues due to the expression of VDRs and vitamin D metabolizing enzymes. These tissues, including the urinary tract and vagina, exhibit responsiveness to and metabolic capabilities for vitamin D [19]. Notably, VDRs play a role in regulating the development, differentiation, and protection of the urinary tract and vaginal epithelium, with vitamin D promoting barrier integrity, upregulating genes encoding epithelial cell junction proteins, and stimulating vaginal epithelium proliferation [20,21,22].

Many studies examined the effects of both oral and vaginal vitamin D on vaginal health of postmenopausal women [23,24,25,26,27,28]. Despite the promising potential, investigations into the effects of vitamin D supplementations on vaginal atrophy in postmenopausal women have yielded inconsistent and inconclusive results [29,30]. The influence of menopause on the pharmacokinetics of vaginal dosage forms adds complexity to these findings [31]. Additionally, factors like dosage regimens, treatment duration, vitamin D type, and lifestyle practices have been identified as predictors of vitamin D status in postmenopausal women [32]. This review summarizes existing evidence on the effects and pharmacological actions of vitamin D on the vaginal, sexual, and urological functions in postmenopausal women and addresses potential clinical uses.

## 2. Methodology

This study constitutes a narrative review focusing on the effects of vitamin D on urogenital health and functions, specifically in postmenopausal women. The review presents a comprehensive analysis of the existing literature, aiming to provide insights into the potential role of vitamin D in maintaining urogenital health during the postmenopausal stage. We searched PubMed and Web of Science using the following keywords: menopause, postmenopausal, vitamin D, cholecalciferol, calcifediol, vagina, sexual, and urinary tract infections alone or in combination. The search encompassed all available literature without language restrictions from the earliest date of publication to the present. The inclusion criteria for selecting the relevant studies comprised original investigations, case studies, systematic reviews, meta-analyses, and expert reviews on vitamin D efficacy outcomes published as full-text articles. A qualitative synthesis was performed to analyze the data, and a narrative synthesis was conducted to provide an overview of the results. The findings were thoroughly scrutinized and discussed, considering the quality of the evidence, the studies’ strengths and limitations, and their implications for clinical practice. The final synthesis was used to develop evidence-based conclusions and recommendations for future research.

## 3. The Pharmacology of Vitamin D

### 3.1. Vitamin D Synthesis

Vitamin D is a unique nutrient that can be synthesized by the body with the help of sunlight or obtained through dietary sources. This essential vitamin is crucial for various bodily functions, including bone health, immune system regulation, and cellular function. Vitamin D synthesis primarily occurs in the skin when exposed to ultraviolet B (UVB) radiation from sunlight. The process begins with 7-dehydrocholesterol, a precursor molecule present in the skin. When UVB rays penetrate the skin, they interact with 7-dehydrocholesterol, triggering a photochemical non-enzymatic reaction that converts it into pre-vitamin D_3_ [33,34]. This pre-vitamin D_3_ is rapidly converted into vitamin D_3_, also known as cholecalciferol, through a thermal isomerization reaction [33,35]. UVB intensity and skin pigmentation level contribute to the rate of D_3_ produced [36]. Vitamin D_3_ can also be obtained through dietary sources or fortified dairy products [37].

### 3.2. Vitamin D Metabolism

Cytochrome P450 mixed-function oxidases (CYPs) perform the crucial steps of 25-hydroxylation, 1-α hydroxylation, and 24-hydroxylation in the metabolism of vitamin D_3_. The initial conversion takes place in the liver, where the enzyme 25-hydroxylase hydroxylates vitamin D_3_ to produce 25-hydroxyvitamin D_3_, also known as calcidiol [35]. This metabolite serves as the primary circulating form of vitamin D and serves as an indicator of the body’s vitamin D status [38]. The liver is recognized as the primary site for 25-hydroxyvitamin D_3_ production. After synthesis in the liver, calcidiol is transported to the kidneys. In the kidneys, the enzyme 1-α-hydroxylase carries out the subsequent hydroxylation, leading to the formation of the active metabolite 1,25-(OH)_2_D_3_ or calcitriol. It has been found that other tissues can also contribute to the production of 1,25-(OH)_2_D_3_. Epithelial cells in the skin, lungs, breasts, testes, ovaries, and placenta have been identified as alternative sources of 1,25-dihydroxyvitamin D_3_ production [39]. It is noteworthy that while liver metabolism requires four enzymes for 25-hydroxylation [40], only one enzyme, namely CYP27B1, has been recognized to possess 25OHD 1α-hydroxylase activity [41,42,43]. Calcitriol binds to vitamin D receptors (VDRs) present in various tissues and cells throughout the body. This binding initiates a cascade of physiological responses, enabling the biological effects of vitamin D to take place.

### 3.3. Vitamin D Mechanism of Action

Extensive research has established that the biological effects of 1,25(OH)D_3_ is mediated through specific changes in gene expression facilitated by the intracellular VDRs [44]. The discovery of numerous VDR binding sites, which regulate hundreds of genes, has intensified interest in understanding the impact of vitamin D on various biological processes. 1,25-dihydroxyvitamin D exerts control over 200 genes, both directly and indirectly. These genes are involved in various crucial cellular processes, such as regulating cellular growth, development, programmed cell death, and forming new blood vessels [38,45]. Upon interaction with 1,25(OH)_2_D_3_, the VDR is activated, leading to its rapid binding to regulatory regions of target genes. This interaction initiates the formation of large protein complexes, which play a crucial role in modulating transcriptional changes [46]. These protein complexes orchestrate the expression of networks of target genes in most target cells, giving rise to specific biological responses. These responses are tissue-specific and encompass a wide range of actions, from complex mechanisms involved in maintaining mineral metabolism homeostasis to focal actions that regulate the growth, differentiation, and functional activity of various cell types, including immune cells, skin cells, pancreatic cells, and bone cells [47].

### 3.4. Predictors of Vitamin D Status

Numerous interventional studies have shed light on the factors that influence the response to vitamin D supplementations. These studies have revealed that factors such as body mass, baseline serum 25(OH)D levels, type of vitamin D supplementation, and the season of the year play significant roles in predicting the impact of vitamin D supplementation on its status [48,49,50,51,52,53,54]. Lower baseline serum 25(OH)D concentrations were associated with a better response to vitamin D supplementation [50,55,56,57,58]. Vitamin D_3_ is superior to vitamin D_2_ in improving vitamin D status [59,60,61,62,63]. Active vitamin D metabolite 25(OH)D_3_ supplementation is more effective than vitamin D_3_ [64,65]. Vitamin D-fortified foods can be an excellent source of increasing serum 25(OH)D, especially when sun exposure is limited. Moreover, melanin in the skin, clothing, and use of sunscreen blocks UVB and thus limits the production of D_3_. Individuals living in latitudes farther from the equator or those with darker skin require more sunlight exposure to synthesize sufficient amounts of vitamin D compared to those living in closer proximity to the equator or individuals with lighter skin [66]. Research indicates that African American women exhibit comparatively lower serum 25(OH)D levels in comparison to their white counterparts [67,68,69,70,71]. Despite this observed disparity in 25(OH)D levels among black women, an intriguing hypothesis has emerged, suggesting that their levels are, in fact, adequate. This hypothesis gains support from the fact that African American women demonstrate lower occurrences of fractures and osteoporosis [70,72,73,74]. Moreover, research conducted through genome-wide association studies has demonstrated that an individual’s genetic makeup also affects their serum vitamin D levels [75].

### 3.5. Effect of Estrogen on Vitamin D Metabolism in Postmenopausal Women

It has been suggested that oral estrogen therapy may increase levels of synthesis of 1,25(OH)D in the kidney in postmenopausal women [76,77,78,79]. It was observed that estrogen increases the free and total calcitriol as well as vitamin D binding protein (DBP) in postmenopausal women [76]. Dick et al. reported that total calcitriol rose only with oral estrogen and not with transdermal. However, free calcitriol was not affected [80]. The observed effects are mainly mediated indirectly through stimulation in 1-α hydroxylase in the kidney [77]. On the other hand, estrogen did not show a significant effect on serum total 25(OH)D over 48 months of treatment with both oral and transdermal estrogen, but a significant increase in free 25(OH)D was observed with transdermal estradiol [81]. This suggests that the effect of estrogen on vitamin D metabolism may be transient. Unlike other studies, Santoro et al. found a significant decrease in DBP with both transdermal and oral estrogen [81]. These findings highlight the complex relationship between estrogen and vitamin D status in light of the variations in measurement parameters and the route of estrogen administration. This effect of estrogen may be limited over an extended period of treatment.

## 4. Genitourinary Syndrome of Menopause

### 4.1. Urogenital Changes in Postmenopausal Women

Urogenital tissues rely on the presence of estrogen to uphold normal physiological functions [82]. Estrogen receptor α is predominantly found in both premenopausal and postmenopausal women, while estrogen receptor β seems to be expressed exclusively in premenopausal women [83]. As women enter the postmenopausal stage, the number of estrogen receptors continues to decline. Estrogen plays a crucial role as a vasoactive hormone, promoting increased blood flow to the urogenital region [84]. This heightened blood flow is essential for maintaining proper vaginal lubrication. Additionally, activated estrogen receptors stimulate epithelial cell proliferation in the vulvovaginal tissues. However, during menopause, these vital functions become compromised. The decline in estrogen levels leads to diminished collagen, elastin, and hyaluronic acid content in the urogenital tissues. Consequently, the epithelium becomes thinner, and vascularity decreases, making postmenopausal women more susceptible to vulvovaginal symptoms [85].

GSM is mostly diagnosed when the patient presents with dyspareunia secondary to vaginal dryness. Improvement in sexual symptoms is closely related to improvement in vaginal symptoms. Dyspareunia resulting from vaginal dryness in postmenopausal women can cause sexual dysfunction [86]. The dryness in the vaginal epithelium can lead to ulceration and fissures during sexual intercourse, causing pain and bleeding [1]. Low estrogen levels also may result in a decline in the activity of brain areas related to sexual arousal [87]. Several surveys have documented the prevalence of sexual complications among peri- and postmenopausal women. There was a decline in sexual activity and frequency, low sexual desire, decreased arousal, and a significant increase in complaints of painful sexual intercourse [12,88,89,90].

Moreover, epidemiological data has shown that estrogen deficiency plays a role in the pathogenesis of urinary tract infections in postmenopausal women, although the underlying mechanism is not elusive [91,92]. It was found that postmenopausal women with a heavy growth of lactobacillus (defined as >10 colonies in the third streak when plated on agar) were less likely to have vaginal colonization with *E.coli,* the most commonly associated with encounter urinary tract infections (UTIs) compared to those with light lactobacillus [93]. Interestingly, literature has shown a strong association between vaginal lactobacilli and the use of hormone replacement therapy [94].

### 4.2. Hormonal Therapy for GSM Symptoms

The approach to addressing GSM is tailored to the intensity of the symptoms experienced. The preferred course of action involves the utilization of low-dose vaginal estrogen therapy, considered the benchmark in treatment. This method is both effective and secure for the majority of patients. It facilitates a rapid renewal of the vaginal epithelium and associated blood vessels, enhances vaginal secretions, reduces vaginal pH levels, reinstates a balanced vaginal microbial environment, and provides relief from comprehensive vulvovaginal symptoms. Notably, this therapeutic approach has demonstrated a remarkable reduction in the susceptibility to UTIs while also mitigating urinary symptoms like urgency and frequency [3,95,96,97,98]. Systematic hormone replacement therapy (HRT) is the preferred approach for patients experiencing vasomotor symptoms, whereas patients with vulvovaginal symptoms alone are usually recommended to use local estrogen therapy [99,100]. No observed difference in breast, endometrial, or cardiovascular risk between the administration of both oral and transdermal HRT was reported [101]. However, benefit/risk assessment should always be carried out when considering systematic hormone therapy [99,100].

### 4.3. Vitamin D Supplementations for GSM Symptoms

Menopause is a crucial turning point in a woman’s life that brings about significant changes, including alterations in vitamin D requirements. Among the various groups affected by vitamin D deficiency, postmenopausal women are particularly susceptible due to a combination of factors. These factors include changes in body composition, advancing age, racial disparities, limited exposure to sunlight, inadequate dietary intake of vitamin D, and increased adiposity [37,102,103,104,105]. Multiple studies have contributed to a growing body of evidence highlighting the connection between vitamin D deficiency and various health conditions experienced during menopause [37,102,103,104,105]. Additionally, the decline in estrogen levels during menopause can lead to vaginal atrophy or GSM, causing symptoms like dryness, itching, and pain during intercourse. Research has suggested that vitamin D deficiency might contribute to the development or worsening of these conditions [25,29,106]. Sexual dysfunction is another issue that can arise during the menopausal transition. Jalali-Chimeh et al. found a potential link between low vitamin D levels and sexual dysfunction among postmenopausal women [16].

## 5. Therapeutic Effects of Vitamin D on Urogenital Functions

### 5.1. Cellular Effects of Vitamin D on Urogenital Tissues

**Figure 1:**
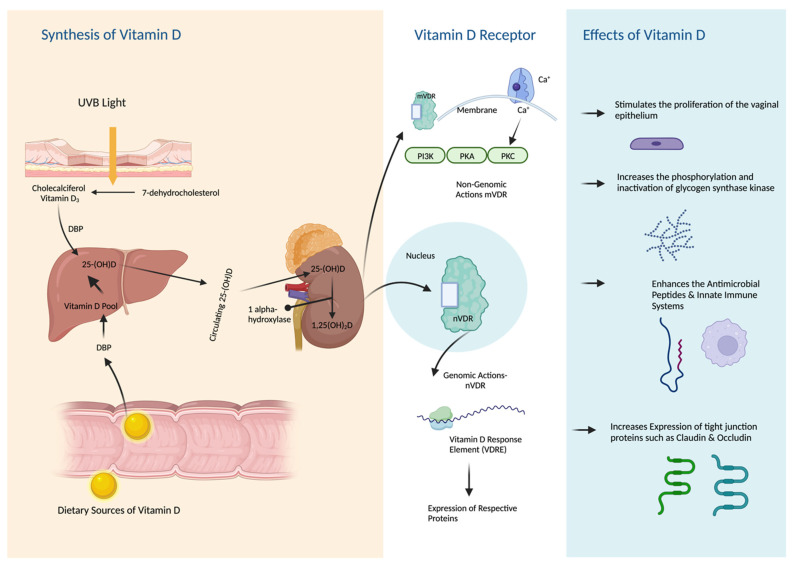
Synthesis, Metabolism, and Effects of Vitamin D on Urogenital Tissues.

Menopause represents an important transition in vitamin D requirements due to the dependence of the VDRs on estrogen [107]. Urogenital organs expressing VDRs are sensitive to changes in vitamin D levels [19]. Vitamin D was shown to affect the activity of VDRs in the vaginal tissues. VDRs are involved in regulating the development, differentiation, and protection of the epithelium of the urinary tract and the vagina [20,21]. Vitamin D was shown to increase the expression of the protein cornifin beta, a marker of squamous differentiation, and upregulates genes encoding epithelial cell junction proteins, promotes the barrier integrity of the vagina, and stimulates the proliferation of the vaginal epithelium [15,108]. Vitamin D also plays a role in the regulation of Ezrin protein [108]. Ezrin, in turn, controls actin-binding proteins that are responsible for interactions between the plasma membrane and cell-to-cell junctions. Ezrin is prominently expressed in the vaginal wall, contributing to the strength and flexibility of the tubular structure. Through activation of the VDR/p-RhoA/p-Ezrin pathway, vitamin D stimulates the proliferation of the vaginal epithelium, resulting in enhanced cell-to-cell junction [106,109]. This robust vaginal wall helps regulate the microbial environment within the vagina, including pH levels and flexibility [110]. In support of this, Lee et al. conducted experiments on a vaginal cell line and human vaginal tissue samples, demonstrating that vitamin D induces the expression of RhoA and Ezrin proteins in vaginal tissue. This induction leads to increased vaginal re-epithelization, comparable to the effects observed with estrogen use [15]. Figure 1. Depicts a simple illustration of vitamin D synthesis, metabolism, and effects on urogenital tissues.

### 5.2. The Effects of Vitamin D on Vaginal Epithelium and pH

**Figure 2:**
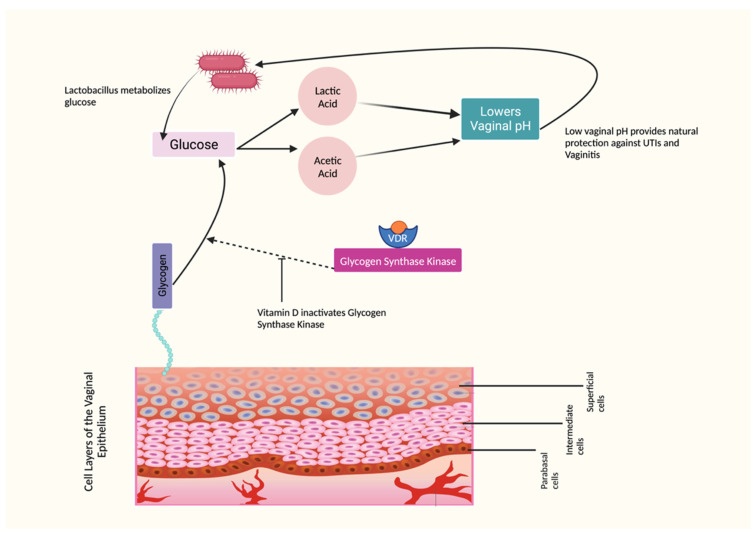
Effects of Vitamin D on Vaginal pH.

The vaginal tissues consist of a nonkeratinized stratified squamous epithelium consisting of superficial, intermediate, and basal cell layers. One of its key functions is to store glycogen, which is converted to glucose. Lactobacillus, a beneficial bacteria, metabolizes this glucose into lactic acid and acetic acid, effectively maintaining a healthy vaginal pH within the range of 3.5–4.5. However, this entire pathway can be disrupted when estrogen levels are low [1,91]. Studies have demonstrated that vitamin D increases the phosphorylation and inactivation of glycogen synthase kinase that inhibits glycogen synthesis. This mechanism is particularly relevant in vaginal health. When vitamin D levels are sufficient, the vaginal glucose balance is positively influenced. This leads to an increase in glycogen deposition, as vitamin D promotes glycogen storage. [22,23,25]. Figure 2 illustrates the role of vitamin D in maintaining balanced vaginal pH.

### 5.3. The Effects of Vitamin D on Vaginal Symptoms

The effects of vitamin D supplementations were a subject of a few trials which examined the effects of oral vitamin D [21,24,26,27,28,111] and topical vitamin D vaginal suppositories and creams [25]. In a recent study, the effects of a vaginal cream containing 1000 IU of vitamin D and 100 IU of vitamin E per dose were examined. The participants applied the cream daily for two weeks and three times a week for an additional 10 weeks. After four weeks of treatment, an improvement was observed in vaginal dryness, itching, and burning [112]. It is worth noting that since this cream was a combination of both vitamin D and vitamin E, the observed effects could be synergistic, with both vitamins contributing to the improvements, in particular, that vitamin E has also shown potential in improving vulvovaginal symptoms associated with GSM in few other studies [113]. The work of Rad et al. found that vitamin D vaginal suppositories over eight weeks showed significant improvements in the superficial, intermediate, and parabasal cell types of the vaginal epithelium. Additionally, there was a significant decrease in vaginal pH [25]. Oral vitamin D supplementations significantly affected the differentiation of superficial, basal, and parabasal cells. However, no significant improvements were observed in vaginal pH [23,28,111]. Similar results were observed in other trials in terms of vaginal pH [24,26,27]. While there were positive findings, such as increased superficial cell count and improved 25(OH)D serum levels, the overall impact on vaginal pH, vaginal dryness, and vaginal atrophy remains inconclusive.

### 5.4. The Effects of Vitamin D on Vaginal Infections

Lactobacilli, yeasts, and bacterial vaginosis-associated bacteria are less commonly parts of the vaginal microflora in postmenopausal women who are not receiving estrogen replacement therapy. This could explain the decrease in the incidence of bacterial vaginosis and yeast vaginitis among these women [114,115]. Vulvovaginal candidiasis (VVC) more commonly occurs at lower pH, which is not present in postmenopausal women due to hypoestrogenism. Clinical observations have reported that postmenopausal women have VVC and candida albicans rarely isolated from vaginal tissues [116]. Interestingly, many studies suggested that estrogen or hormone replacement therapy plays a critical role in increasing the susceptibility of acquiring VVC in postmenopausal women [94,116,117,118]. The proposed mechanism is an interaction between estradiol and the estrogen-binding protein in yeast [119]. On the other hand, very few studies looked at the prevalence of bacterial vaginosis (BV) in postmenopausal women. According to Bodnar et al., there was a relatively high incidence of BV among women with a serum 25(OH)D concentration below 20 nmol/L, while the prevalence was much lower among women with a serum 25(OH)D concentration exceeding 80 nmol/L. The study also highlighted a dose-response relationship between the level of 25(OH)D and the occurrence of BV [120]. However, Kaur et al. found no association between serum vitamin D levels and BV [121]. Ginkel et al. concluded that women on estrogen replacement therapy are less likely to have vaginal colonization with anaerobic bacteria and that estrogen may potentiate the effects of lactobacilli on vaginal pH [122].

### 5.5. The Effects of Vitamin D on Sexual Functions

It was demonstrated by a few studies that there is an association between vitamin D deficiency and a decline in sexual functions, including sexual desire, orgasm, and satisfaction in women [17,123]. Small clinical trials demonstrated improved female sexual function with vitamin D supplementation. Although some of these studies were conducted on young premenopausal women, this improvement was supported by the fact that VDRs are present in the uterus and ovaries and may have a role in sexual function. Also, data has shown that symptom severity was correlated with vitamin D serum levels [16,123,124].

A three-arm randomized-blind clinical trial included postmenopausal sexually active women to test the effects of vitamin D vaginal suppositories on sexual function. Women were administered vitamin D suppositories (1000 IU) for eight weeks. The treatment group showed statistically significant changes in sexual function compared to the control group. However, looking at the scores in the intervention group over the follow-up period, this improvement was of minimal clinical significance. Surprisingly, after two months of treatment, sexual function dropped to below baseline levels [30]. A different study investigated how a combination of isoflavones, calcium, vitamin D, and inulin affects the sexual functioning of postmenopausal women. The findings revealed an improvement in sexual functions as measured by the Female Sexual Function Index (FSFI) after 12 months of treatment. However, it is difficult to determine whether the observed effect was solely due to the individual ingredients or if they worked together synergistically. Nonetheless, it is worth noting that serum vitamin D levels increased after 12 months. It is still difficult to specifically attribute its effects. Additionally, it is important to consider that the study had a limited sample size and lacked a direct comparison between the treatment group and the control group, affecting the findings’ overall reliability [125]. A combined vaginal vitamin D and E cream improved libido, orgasm, and sexual frequency after only 4 weeks of application [112].

### 5.6. The Role of Vitamin D in UTIs: Effects on the Immune Function

The significance of vitamin D, specifically 1,25(OH)D_2_ and its metabolites have become evident in immune function following the discovery of VDR expression in activated inflammatory cells [126,127]. Changes in the immune system and increased pro-inflammatory serum marker levels, cytokine responses in body cells, decreased CD4 T and B lymphocyte levels, and natural killer cell cytotoxic activity are all observed post-menopause [21]. Various protective factors, including antimicrobial peptides and the innate immune system, play a role in preventing UTIs. Vitamin D plays a supportive and enhancing role in these systems. Research indicates that vitamin D stimulates the production of cathelicidin in the urinary bladder [18,20,128].

### 5.7. The Role of Vitamin D in UTIs: Effects on Tight Junction Proteins

The role of vitamin D in tight junction proteins in the urinary tract remains relatively unexplored. Nonetheless, studies have shown that uropathogenic Escherichia coli infection disrupts the tight junction barrier by downregulating occludin and claudins in bladder epithelial cells [129]. Strengthening the urothelial barrier to prevent infections becomes an appealing approach. It is suggested that vitamin D deficiency decreases the expression of occludin and claudin-5 [130]. Moreover, the VDR was demonstrated to mediate the protective effect of vitamin D-induced expression of occludin, claudin-5, and zonula occludens [131].

### 5.8. The Effects of Vitamin D on Pelvic Floor Disorders

Difficulty in urination, hesitancy, delay in urination, dyspareunia, and vaginal prolapse are common in postmenopausal women and are attributed to pelvic floor dysfunction (PFD) [132]. A double-blinded controlled trial examining the effects of vaginal vitamin D on women with PFDs, including pelvic organ prolapse and urinary and fecal incontinence, found that women with PFDs had lower mean vitamin D levels than otherwise healthy postmenopausal women. However, the association was not statistically significant. Vitamin D deficiency was shown to increase the risk of overactive bladder (OAB) and urinary incontinence [133]. Thus, there is growing recognition of the role of vitamin D supplementation in reducing these risks. A study conducted on postmenopausal women with stress incontinence reported a positive effect with a combination of high-dose vitamin D and estriol. After six weeks of treatment with high-dose vaginal suppositories, a significant reduction in the risk of OAB onset was observed [134]. After six weeks of treatment with high-dose vaginal suppositories, a positive and significant effect was observed where higher intakes of vitamin D decreased the risk of OAB onset [135]. Similarly, a weekly dose of 50,000 IU vitamin D for eight weeks was shown to reduce the severity of UI and frequency of nocturia in postmenopausal women [136]. The VDR has also been identified in the detrusor wall; thus, its insufficient level may impact bladder function and pelvic floor muscle weakness. Vitamin D may play a role in the efficiency of muscle function that is distinct from the role of calcium in muscle contractility [123,124,132].

## 6. Vaginal Dosage Form as Route of Delivery

As vitamin D is available in oral and vaginal dosage forms, the pharmacokinetics of the administered form should be considered. When the drug is intended for local effects, such as treating vulvovaginal symptoms, it must be distributed and absorbed into the vaginal epithelium. The major mechanisms of transport across vaginal epithelium include transcellular transport and receptor-mediated transcytosis [31]. In postmenopausal women, the physiological changes in the vagina can affect vaginal uptake. Vaginal thickness, vaginal secretions, local microflora, vaginal pH, and sexual arousal are all altered and can affect drug uptake. Physiochemical properties of the drug also have an effect. It has been demonstrated that vaginal permeability is much greater for lipophilic steroids [137]. Sensory factors such as packaging, product shape, size, and firmness have been reported to affect women’s willingness to try vaginal dosage forms [138,139]. Such acceptability and factors may differ based on the intended use or geographical region and culture [140].

## 7. Future Research Directions

Recognizing the pivotal role of vitamin D supplementation demands attention. Emerging research presents encouraging evidence of its potential to positively impact sexual functioning and urogenital health among postmenopausal women. However, the existing landscape reveals a lack of robust randomized controlled trials marked by variations in participant demographics, dosing regimens, intervention durations, and assessment protocols. This heterogeneity challenges the establishment of definitive conclusions regarding the efficacy of vitamin D supplementation.

To bridge these research gaps, future studies should prioritize comprehensive investigations that address specific aspects. Firstly, well-designed randomized controlled trials with consistent methodologies are necessary to offer conclusive insights. Standardization of dosage, intervention duration, and outcome assessment would enhance the comparability of results across studies. Secondly, combining objective measurements like the vaginal maturation index with subjective self-assessments of sexual functioning could provide a more nuanced understanding of vitamin D’s effects. This integrated approach would ensure a more comprehensive evaluation of the potential benefits.

**Figure 3:**
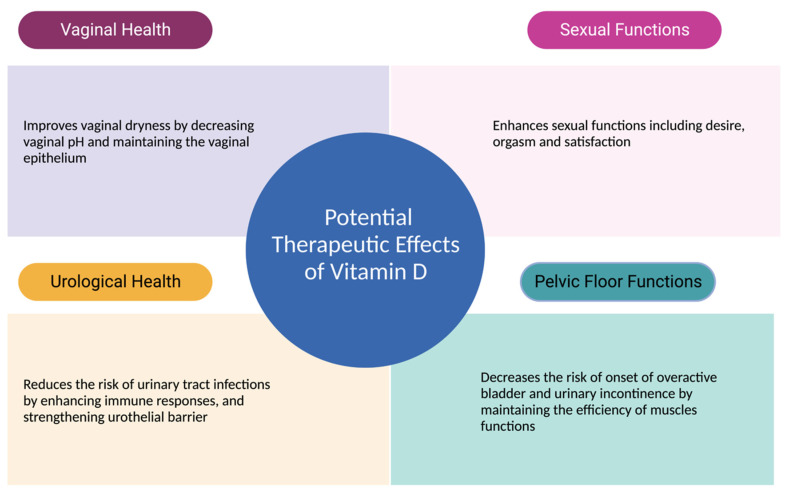
Therapeutic Effects of Vitamin D Urogenital Functions.

Such advancements in research would significantly benefit both clinicians and postmenopausal women in terms of health and well-being. By establishing evidence-based guidelines grounded in rigorous research, clinicians can confidently tailor interventions to address individual needs. Specific dosing recommendations, intervention durations, and expected outcomes could be established, leading to more effective and personalized care strategies. For postmenopausal women, this translates into improved urogenital health, enhanced sexual well-being, and an overall better quality of life. Clearer insights from well-structured studies would empower women to make informed decisions about their health and provide them with the means to actively participate in their own care. Figure 3 summarizes all potential therapeutic effects of vitamin D on urogenital health and sexual functions.

## 8. Conclusions

The reviewed literature provided valuable insights into the potential therapeutic effects of vitamin D on various aspects of women’s health, specifically related to vaginal symptoms, urogenital infections, sexual functioning, and pelvic floor disorders in postmenopausal women. While the findings are not consistently conclusive, they suggest that vitamin D supplementation, whether topically or orally, may offer benefits in improving vaginal symptoms and sexual function and potentially reducing the risk of UTIs. However, further research is necessary to establish optimal dosage, treatment duration, and long-term effects. Nonetheless, these findings contribute to our understanding of the potential role of vitamin D in promoting women’s health and highlight avenues for future investigation and clinical applications.

## 9. Key Findings

- The role of vitamin D on urogenital and sexual health in postmenopausal women revealed a limited but growing body of research.
- Vitamin D has the potential to support postmenopausal women’s urogenital and sexual health.
- Vitamin D receptors play a significant role in mediating and maintaining the pharmacological effects of vitamin D on urogenital organs.
- Both oral and vaginal vitamin D were shown to improve vulvovaginal symptoms, sexual functioning and reduce the risk of urinary tract infections.
- Further research is needed to fully understand the therapeutic effects of vitamin D supplementation on urogenital and sexual health in postmenopausal women.

## Data Availability

Not applicable.

## References

[1] Gandhi J., Chen A., Dagur G., Suh Y., Smith N., Cali B., Khan S.A. (2016). Genitourinary syndrome of menopause: An overview of clinical manifestations, pathophysiology, etiology, evaluation, and management. Am. J. Obstet. Gynecol..

[2] North American Menopause Society (2013). Management of symptomatic vulvovaginal atrophy: 2013 position statement of The North American Menopause Society. Menopause.

[3] Angelou K., Grigoriadis T., Diakosavvas M., Zacharakis D., Athanasiou S. (2020). The Genitourinary Syndrome of Menopause: An Overview of the Recent Data. Cureus.

[4] Faubion S.S., Sood R., Kapoor E. (2017). Genitourinary Syndrome of Menopause: Management Strategies for the Clinician. Mayo Clin. Proc..

[5] Hugenholtz F., van der Veer C., Terpstra M.L., Borgdorff H., van Houdt R., Bruisten S., Geerlings S.E., van de Wijgert J. (2022). Urine and vaginal microbiota compositions of postmenopausal and premenopausal women differ regardless of recurrent urinary tract infection and renal transplant status. Sci. Rep..

[6] Nappi R.E., Martini E., Cucinella L., Martella S., Tiranini L., Inzoli A., Brambilla E., Bosoni D., Cassani C., Gardella B. (2019). Addressing Vulvovaginal Atrophy (VVA)/Genitourinary Syndrome of Menopause (GSM) for Healthy Aging in Women. Front. Endocrinol..

[7] Portman D.J., Gass M.L., Vulvovaginal Atrophy Terminology Consensus Conference Panel (2014). Genitourinary syndrome of menopause: New terminology for vulvovaginal atrophy from the International Society for the Study of Women’s Sexual Health and the North American Menopause Society. Menopause.

[8] Domoney C., Short H., Particco M., Panay N. (2020). Symptoms, attitudes and treatment perceptions of vulvo-vaginal atrophy in UK postmenopausal women: Results from the REVIVE-EU study. Post Reprod. Health.

[9] Krychman M., Graham S., Bernick B., Mirkin S., Kingsberg S.A. (2017). The Women’s EMPOWER Survey: Women’s Knowledge and Awareness of Treatment Options for Vulvar and Vaginal Atrophy Remains Inadequate. J. Sex. Med..

[10] Moral E., Delgado J.L., Carmona F., Caballero B., Guillan C., Gonzalez P.M., Suarez-Almarza J., Velasco-Ortega S., Nieto C., as the writing group of the GENISSE study (2018). Genitourinary syndrome of menopause. Prevalence and quality of life in Spanish postmenopausal women. The GENISSE study. Climacteric.

[11] Nappi R.E., de Melo N.R., Martino M., Celis-Gonzalez C., Villaseca P., Rohrich S., Palacios S. (2018). Vaginal Health: Insights, Views & Attitudes (VIVA-LATAM): Results from a survey in Latin America. Climacteric.

[12] Nappi R.E., Kingsberg S., Maamari R., Simon J. (2013). The CLOSER (CLarifying Vaginal Atrophy’s Impact On SEx and Relationships) survey: Implications of vaginal discomfort in postmenopausal women and in male partners. J. Sex. Med..

[13] Palma F., Volpe A., Villa P., Cagnacci A., as the writing group of the AGATA study (2016). Vaginal atrophy of women in postmenopause. Results from a multicentric observational study: The AGATA study. Maturitas.

[14] Mac Bride M.B., Rhodes D.J., Shuster L.T. (2010). Vulvovaginal atrophy. Mayo Clin. Proc..

[15] Lee A., Lee M.R., Lee H.H., Kim Y.S., Kim J.M., Enkhbold T., Kim T.H. (2017). Vitamin D Proliferates Vaginal Epithelium through RhoA Expression in Postmenopausal Atrophic Vagina tissue. Mol. Cells.

[16] Jalali-Chimeh F., Gholamrezaei A., Vafa M., Nasiri M., Abiri B., Darooneh T., Ozgoli G. (2019). Effect of Vitamin D Therapy on Sexual Function in Women with Sexual Dysfunction and Vitamin D Deficiency: A Randomized, Double-Blind, Placebo Controlled Clinical Trial. J. Urol..

[17] Askin M., Koc E.M., Soyoz M., Aksun S., Aydogmus S., Sozmen K. (2019). Relationship between Postmenopausal Vitamin D Level, Menopausal Symptoms and Sexual Functions. J. Coll. Physicians Surg. Pak..

[18] Hertting O., Holm A., Luthje P., Brauner H., Dyrdak R., Jonasson A.F., Wiklund P., Chromek M., Brauner A. (2010). Vitamin D induction of the human antimicrobial Peptide cathelicidin in the urinary bladder. PLoS ONE.

[19] Lorenzen M., Boisen I.M., Mortensen L.J., Lanske B., Juul A., Blomberg Jensen M. (2017). Reproductive endocrinology of vitamin D. Mol. Cell. Endocrinol..

[20] Skowronska P., Pastuszek E., Kuczynski W., Jaszczol M., Kuc P., Jakiel G., Woclawek-Potocka I., Lukaszuk K. (2016). The role of vitamin D in reproductive dysfunction in women—A systematic review. Ann. Agric. Environ. Med..

[21] Kamronrithisorn T., Manonai J., Vallibhakara S.A., Sophonsritsuk A., Vallibhakara O. (2020). Effect of Vitamin D Supplement on Vulvovaginal Atrophy of the Menopause. Nutrients.

[22] Jefferson K.K., Parikh H.I., Garcia E.M., Edwards D.J., Serrano M.G., Hewison M., Shary J.R., Powell A.M., Hollis B.W., Fettweis J.M. (2019). Relationship between vitamin D status and the vaginal microbiome during pregnancy. J. Perinatol..

[23] Yildirim B., Kaleli B., Duzcan E., Topuz O. (2004). The effects of postmenopausal Vitamin D treatment on vaginal atrophy. Maturitas.

[24] Saeideh Z., Raziyeh M., Soghrat F. (2010). Comparing the effects of continuous hormone replacement therapy and tibolone on the genital tract of menopausal women; a randomized controlled trial. J. Reprod. Infertil..

[25] Rad P., Tadayon M., Abbaspour M., Latifi S.M., Rashidi I., Delaviz H. (2015). The effect of vitamin D on vaginal atrophy in postmenopausal women. Iran J. Nurs. Midwifery Res..

[26] Mucci M., Carraro C., Mancino P., Monti M., Papadia L.S., Volpini G., Benvenuti C. (2006). Soy isoflavones, lactobacilli, Magnolia bark extract, vitamin D3 and calcium. Controlled clinical study in menopause. Minerva Ginecol..

[27] Checa M.A., Garrido A., Prat M., Conangla M., Rueda C., Carreras R. (2005). A comparison of raloxifene and calcium plus vitamin D on vaginal atrophy after discontinuation of long-standing postmenopausal hormone therapy in osteoporotic women. A randomized, masked-evaluator, one-year, prospective study. Maturitas.

[28] Bala R., Kaur H., Nagpal M. (2016). Authenticity of vitamin D in modified vaginal health index in geriatric subjects. Int. J. Reprod. Contracept. Obstet. Gynecol..

[29] Riazi H., Ghazanfarpour M., Taebi M., Abdolahian S. (2019). Effect of Vitamin D on the Vaginal Health of Menopausal Women: A Systematic Review. J. Menopausal. Med..

[30] Sarebani Z., Chegini V., Chen H., Aali E., Mirzadeh M., Abbaspour M., Griffiths M.D., Alimoradi Z. (2023). Effect of vitamin D vaginal suppository on sexual functioning among postmenopausal women: A three-arm randomized controlled clinical trial. Obstet. Gynecol. Sci..

[31] Hussain A., Ahsan F. (2005). The vagina as a route for systemic drug delivery. J. Control. Release.

[32] Hassanein M.M., Huri H.Z., Baig K., Abduelkarem A.R. (2023). Determinants and Effects of Vitamin D Supplementation in Postmenopausal Women: A Systematic Review. Nutrients.

[33] Tian X.Q., Chen T.C., Matsuoka L.Y., Wortsman J., Holick M.F. (1993). Kinetic and thermodynamic studies of the conversion of previtamin D3 to vitamin D3 in human skin. J. Biol. Chem..

[34] Holick M.F. (2003). Vitamin D: A millenium perspective. J. Cell. Biochem..

[35] Bikle D.D. (2014). Vitamin D metabolism, mechanism of action, and clinical applications. Chem. Biol..

[36] Holick M.F., MacLaughlin J.A., Clark M.B., Holick S.A., Potts J.T., Anderson R.R., Blank I.H., Parrish J.A., Elias P. (1980). Photosynthesis of previtamin D3 in human skin and the physiologic consequences. Science.

[37] Holick M.F. (2007). Vitamin D deficiency. N. Engl. J. Med..

[38] Holick M.F. (2009). Vitamin D status: Measurement, interpretation, and clinical application. Ann. Epidemiol..

[39] Bikle D.D. (2007). What is new in vitamin D: 2006–2007. Curr. Opin. Rheumatol..

[40] Cheng J.B., Levine M.A., Bell N.H., Mangelsdorf D.J., Russell D.W. (2004). Genetic evidence that the human CYP2R1 enzyme is a key vitamin D 25-hydroxylase. Proc. Natl. Acad. Sci. USA.

[41] Fu G.K., Lin D., Zhang M.Y., Bikle D.D., Shackleton C.H., Miller W.L., Portale A.A. (1997). Cloning of human 25-hydroxyvitamin D-1 alpha-hydroxylase and mutations causing vitamin D-dependent rickets type 1. Mol. Endocrinol..

[42] Shinki T., Shimada H., Wakino S., Anazawa H., Hayashi M., Saruta T., DeLuca H.F., Suda T. (1997). Cloning and expression of rat 25-hydroxyvitamin D3-1alpha-hydroxylase cDNA. Proc. Natl. Acad. Sci. USA.

[43] Takeyama K., Kitanaka S., Sato T., Kobori M., Yanagisawa J., Kato S. (1997). 25-Hydroxyvitamin D3 1alpha-hydroxylase and vitamin D synthesis. Science.

[44] Haussler M.R., Whitfield G.K., Haussler C.A., Hsieh J.C., Thompson P.D., Selznick S.H., Dominguez C.E., Jurutka P.W. (1998). The nuclear vitamin D receptor: Biological and molecular regulatory properties revealed. J. Bone Miner. Res..

[45] Nagpal S., Na S., Rathnachalam R. (2005). Noncalcemic actions of vitamin D receptor ligands. Endocr. Rev..

[46] Sutton A.L., MacDonald P.N. (2003). Vitamin D: More than a “bone-a-fide” hormone. Mol. Endocrinol..

[47] Bouillon R., Carmeliet G., Verlinden L., van Etten E., Verstuyf A., Luderer H.F., Lieben L., Mathieu C., Demay M. (2008). Vitamin D and human health: Lessons from vitamin D receptor null mice. Endocr. Rev..

[48] Lips P., van Schoor N.M., de Jongh R.T. (2014). Diet, sun, and lifestyle as determinants of vitamin D status. Ann. N. Y. Acad. Sci..

[49] Sollid S.T., Hutchinson M.Y., Fuskevag O.M., Joakimsen R.M., Jorde R. (2016). Large Individual Differences in Serum 25-Hydroxyvitamin D Response to Vitamin D Supplementation: Effects of Genetic Factors, Body Mass Index, and Baseline Concentration. Results from a Randomized Controlled Trial. Horm. Metab. Res..

[50] Whiting S.J., Bonjour J.P., Payen F.D., Rousseau B. (2015). Moderate amounts of vitamin D3 in supplements are effective in raising serum 25-hydroxyvitamin D from low baseline levels in adults: A systematic review. Nutrients.

[51] Gallagher J.C., Yalamanchili V., Smith L.M. (2013). The effect of vitamin D supplementation on serum 25(OH)D in thin and obese women. J. Steroid Biochem. Mol. Biol..

[52] Heaney R.P., Davies K.M., Chen T.C., Holick M.F., Barger-Lux M.J. (2003). Human serum 25-hydroxycholecalciferol response to extended oral dosing with cholecalciferol. Am. J. Clin. Nutr..

[53] Kasahara A.K., Singh R.J., Noymer A. (2013). Vitamin D (25OHD) Serum Seasonality in the United States. PLoS ONE.

[54] Rees J.R., Mott L.A., Barry E.L., Baron J.A., Bostick R.M., Figueiredo J.C., Bresalier R.S., Robertson D.J., Peacock J.L. (2016). Lifestyle and Other Factors Explain One-Half of the Variability in the Serum 25-Hydroxyvitamin D Response to Cholecalciferol Supplementation in Healthy Adults. J. Nutr..

[55] Viljakainen H.T., Palssa A., Karkkainen M., Jakobsen J., Lamberg-Allardt C. (2006). How much vitamin D3 do the elderly need?. J. Am. Coll. Nutr..

[56] Black L.J., Seamans K.M., Cashman K.D., Kiely M. (2012). An updated systematic review and meta-analysis of the efficacy of vitamin D food fortification. J. Nutr..

[57] Bonjour J.P., Dontot-Payen F., Rouy E., Walrand S., Rousseau B. (2018). Evolution of Serum 25OHD in Response to Vitamin D(3)-Fortified Yogurts Consumed by Healthy Menopausal Women: A 6-Month Randomized Controlled Trial Assessing the Interactions between Doses, Baseline Vitamin D Status, and Seasonality. J. Am. Coll. Nutr..

[58] Talwar S.A., Aloia J.F., Pollack S., Yeh J.K. (2007). Dose response to vitamin D supplementation among postmenopausal African American women. Am. J. Clin. Nutr..

[59] Tjellesen L., Hummer L., Christiansen C., Rodbro P. (1986). Serum concentration of vitamin D metabolites during treatment with vitamin D2 and D3 in normal premenopausal women. Bone Miner..

[60] Trang H.M., Cole D.E., Rubin L.A., Pierratos A., Siu S., Vieth R. (1998). Evidence that vitamin D3 increases serum 25-hydroxyvitamin D more efficiently than does vitamin D2. Am. J. Clin. Nutr..

[61] Armas L.A., Hollis B.W., Heaney R.P. (2004). Vitamin D2 is much less effective than vitamin D3 in humans. J. Clin. Endocrinol. Metab..

[62] Tripkovic L., Lambert H., Hart K., Smith C.P., Bucca G., Penson S., Chope G., Hypponen E., Berry J., Vieth R. (2012). Comparison of vitamin D2 and vitamin D3 supplementation in raising serum 25-hydroxyvitamin D status: A systematic review and meta-analysis. Am. J. Clin. Nutr..

[63] Mastaglia S.R., Mautalen C.A., Parisi M.S., Oliveri B. (2006). Vitamin D2 dose required to rapidly increase 25OHD levels in osteoporotic women. Eur. J. Clin. Nutr..

[64] Bischoff-Ferrari H.A., Dawson-Hughes B., Stocklin E., Sidelnikov E., Willett W.C., Edel J.O., Stahelin H.B., Wolfram S., Jetter A., Schwager J. (2012). Oral supplementation with 25(OH)D3 versus vitamin D3: Effects on 25(OH)D levels, lower extremity function, blood pressure, and markers of innate immunity. J. Bone Miner. Res..

[65] Perez-Castrillon J.L., Duenas-Laita A., Brandi M.L., Jodar E., Del Pino-Montes J., Quesada-Gomez J.M., Cereto Castro F., Gomez-Alonso C., Gallego Lopez L., Olmos Martinez J.M. (2021). Calcifediol is superior to cholecalciferol in improving vitamin D status in postmenopausal women: A randomized trial. J. Bone Miner. Res..

[66] Webb A.R., DeCosta B.R., Holick M.F. (1989). Sunlight regulates the cutaneous production of vitamin D3 by causing its photodegradation. J. Clin. Endocrinol. Metab..

[67] Holick M.F. (2006). High prevalence of vitamin D inadequacy and implications for health. Mayo Clin. Proc..

[68] Signorello L.B., Williams S.M., Zheng W., Smith J.R., Long J., Cai Q., Hargreaves M.K., Hollis B.W., Blot W.J. (2010). Blood vitamin d levels in relation to genetic estimation of African ancestry. Cancer Epidemiol. Biomark. Prev..

[69] Aloia J.F., Talwar S.A., Pollack S., Yeh J. (2005). A randomized controlled trial of vitamin D3 supplementation in African American women. Arch. Intern. Med..

[70] Aloia J.F., Vaswani A., Yeh J.K., Flaster E. (1996). Risk for osteoporosis in black women. Calcif. Tissue Int..

[71] Nesby-O’Dell S., Scanlon K.S., Cogswell M.E., Gillespie C., Hollis B.W., Looker A.C., Allen C., Doughertly C., Gunter E.W., Bowman B.A. (2002). Hypovitaminosis D prevalence and determinants among African American and white women of reproductive age: Third National Health and Nutrition Examination Survey, 1988–1994. Am. J. Clin. Nutr..

[72] Barrett-Connor E., Siris E.S., Wehren L.E., Miller P.D., Abbott T.A., Berger M.L., Santora A.C., Sherwood L.M. (2005). Osteoporosis and fracture risk in women of different ethnic groups. J. Bone Miner. Res..

[73] Bryant R.J., Wastney M.E., Martin B.R., Wood O., McCabe G.P., Morshidi M., Smith D.L., Peacock M., Weaver C.M. (2003). Racial differences in bone turnover and calcium metabolism in adolescent females. J. Clin. Endocrinol. Metab..

[74] Harris S.S. (2006). Vitamin D and African Americans. J. Nutr..

[75] Zhang M., Zhao L.J., Zhou Y., Badr R., Watson P., Ye A., Zhou B., Zhang J., Deng H.W., Recker R.R. (2017). SNP rs11185644 of RXRA gene is identified for dose-response variability to vitamin D3 supplementation: A randomized clinical trial. Sci. Rep..

[76] Cheema C., Grant B.F., Marcus R. (1989). Effects of estrogen on circulating “free” and total 1,25-dihydroxyvitamin D and on the parathyroid-vitamin D axis in postmenopausal women. J. Clin. Investig..

[77] Gallagher J.C., Riggs B.L., DeLuca H.F. (1980). Effect of estrogen on calcium absorption and serum vitamin D metabolites in postmenopausal osteoporosis. J. Clin. Endocrinol. Metab..

[78] Bikle D.D., Halloran B.P., Harris S.T., Portale A.A. (1992). Progestin antagonism of estrogen stimulated 1,25-dihydroxyvitamin D levels. J. Clin. Endocrinol. Metab..

[79] Prince R.L. (1994). Counterpoint: Estrogen effects on calcitropic hormones and calcium homeostasis. Endocr. Rev..

[80] Dick I.M., Prince R.L., Kelly J.J., Ho K.K. (1995). Oestrogen effects on calcitriol levels in post-menopausal women: A comparison of oral versus transdermal administration. Clin. Endocrinol..

[81] Santoro A.M., Simpson C.A., Cong E., Haas A., Sullivan R.R., Parziale S., Deng Y., Insogna K.L. (2022). Differing effects of oral conjugated equine estrogen and transdermal estradiol on vitamin D metabolism in postmenopausal women: A 4-year longitudinal study. Menopause.

[82] Goldstein I. (2010). Recognizing and treating urogenital atrophy in postmenopausal women. J. Womens Health.

[83] Chen G.D., Oliver R.H., Leung B.S., Lin L.Y., Yeh J. (1999). Estrogen receptor alpha and beta expression in the vaginal walls and uterosacral ligaments of premenopausal and postmenopausal women. Fertil. Steril..

[84] North American Menopause Society (2007). The role of local vaginal estrogen for treatment of vaginal atrophy in postmenopausal women: 2007 position statement of The North American Menopause Society. Menopause.

[85] Nappi R.E., Palacios S. (2014). Impact of vulvovaginal atrophy on sexual health and quality of life at postmenopause. Climacteric.

[86] Costantino D., Guaraldi C. (2008). Effectiveness and safety of vaginal suppositories for the treatment of the vaginal atrophy in postmenopausal women: An open, non-controlled clinical trial. Eur. Rev. Med. Pharmacol. Sci..

[87] Kim G.W., Jeong G.W. (2017). Menopause-related brain activation patterns during visual sexual arousal in menopausal women: An fMRI pilot study using time-course analysis. Neuroscience.

[88] Avis N.E., Stellato R., Crawford S., Johannes C., Longcope C. (2000). Is there an association between menopause status and sexual functioning?. Menopause.

[89] Dennerstein L., Dudley E., Burger H. (2001). Are changes in sexual functioning during midlife due to aging or menopause?. Fertil. Steril..

[90] Nappi R.E., Nijland E.A. (2008). Women’s perception of sexuality around the menopause: Outcomes of a European telephone survey. Eur. J. Obstet. Gynecol. Reprod. Biol..

[91] Altoparlak U., Kadanali A., Kadanali S. (2004). Correlation of urinary tract infections with the vaginal colonization in postmenopausal women. Mikrobiyol. Bul..

[92] Luthje P., Brauner H., Ramos N.L., Ovregaard A., Glaser R., Hirschberg A.L., Aspenstrom P., Brauner A. (2013). Estrogen supports urothelial defense mechanisms. Sci. Transl. Med..

[93] Pabich W.L., Fihn S.D., Stamm W.E., Scholes D., Boyko E.J., Gupta K. (2003). Prevalence and determinants of vaginal flora alterations in postmenopausal women. J. Infect. Dis..

[94] Raz R., Stamm W.E. (1993). A controlled trial of intravaginal estriol in postmenopausal women with recurrent urinary tract infections. N. Engl. J. Med..

[95] Cellai I., Di Stasi V., Comeglio P., Maseroli E., Todisco T., Corno C., Filippi S., Cipriani S., Sorbi F., Fambrini M. (2021). Insight on the Intracrinology of Menopause: Androgen Production within the Human Vagina. Endocrinology.

[96] Scavello I., Maseroli E., Di Stasi V., Vignozzi L. (2019). Sexual Health in Menopause. Medicina.

[97] Archer D.F., Labrie F., Bouchard C., Portman D.J., Koltun W., Cusan L., Labrie C., Cote I., Lavoie L., Martel C. (2015). Treatment of pain at sexual activity (dyspareunia) with intravaginal dehydroepiandrosterone (prasterone). Menopause.

[98] Palacios S., Mejia A., Neyro J.L. (2015). Treatment of the genitourinary syndrome of menopause. Climacteric.

[99] Baber R.J., Panay N., Fenton A., Group I.M.S.W. (2016). 2016 IMS Recommendations on women’s midlife health and menopause hormone therapy. Climacteric.

[100] North American Menopause Society (2017). The 2017 hormone therapy position statement of The North American Menopause Society. Menopause.

[101] Goldstajn M.S., Mikus M., Ferrari F.A., Bosco M., Uccella S., Noventa M., Torok P., Terzic S., Lagana A.S., Garzon S. (2023). Effects of transdermal versus oral hormone replacement therapy in postmenopause: A systematic review. Arch. Gynecol. Obstet..

[102] Holick M.F., Chen T.C. (2008). Vitamin D deficiency: A worldwide problem with health consequences. Am. J. Clin. Nutr..

[103] Lopez-Baena M.T., Perez-Roncero G.R., Perez-Lopez F.R., Mezones-Holguin E., Chedraui P. (2020). Vitamin D, menopause, and aging: Quo vadis?. Climacteric.

[104] Delle Monache S., Di Fulvio P., Iannetti E., Valerii L., Capone L., Nespoli M.G., Bologna M., Angelucci A. (2019). Body mass index represents a good predictor of vitamin D status in women independently from age. Clin. Nutr..

[105] Feghaly J., Johnson P., Kalhan A. (2020). Vitamin D and obesity in adults: A pathophysiological and clinical update. Br. J. Hosp. Med..

[106] Donders G.G.G., Ruban K., Bellen G., Grinceviciene S. (2019). Pharmacotherapy for the treatment of vaginal atrophy. Expert Opin Pharmacother..

[107] Duque G., El Abdaimi K., Macoritto M., Miller M.M., Kremer R. (2002). Estrogens (E2) regulate expression and response of 1,25-dihydroxyvitamin D3 receptors in bone cells: Changes with aging and hormone deprivation. Biochem. Biophys. Res. Commun..

[108] Abban G., Yildirim N.B., Jetten A.M. (2008). Regulation of the vitamin D receptor and cornifin beta expression in vaginal epithelium of the rats through vitamin D3. Eur. J. Histochem..

[109] Bikle D., Teichert A., Hawker N., Xie Z., Oda Y. (2007). Sequential regulation of keratinocyte differentiation by 1,25(OH)2D3, VDR, and its coregulators. J. Steroid Biochem. Mol. Biol..

[110] Fadiel A., Lee H.H., Demir N., Richman S., Iwasaki A., Connell K., Naftolin F. (2008). Ezrin is a key element in the human vagina. Maturitas.

[111] Carranza-Lira S., Amador-Perez C., Macgregor-Gooch A.L., Estrada-Moscoso I. (2012). Changes in maturation index and vaginal dryness in postmenopausal women who use or not calcitriol. Rev. Med. Inst. Mex. Seguro Soc..

[112] Radnia N., Hosseini S.T., Vafaei S.Y., Pirdehghan A., Mehrabadi N.L. (2023). The effect of conjugated estrogens vaginal cream and a combined vaginal cream of vitamins D and E in the treatment of genitourinary syndrome. J. Fam. Med. Prim. Care.

[113] Porterfield L., Wur N., Delgado Z.S., Syed F., Song A., Weller S.C. (2022). Vaginal Vitamin E for Treatment of Genitourinary Syndrome of Menopause: A Systematic Review of Randomized Controlled Trials. J. Menopausal. Med..

[114] Hillier S.L., Lau R.J. (1997). Vaginal microflora in postmenopausal women who have not received estrogen replacement therapy. Clin. Infect. Dis..

[115] Cauci S., Driussi S., De Santo D., Penacchioni P., Iannicelli T., Lanzafame P., De Seta F., Quadrifoglio F., de Aloysio D., Guaschino S. (2002). Prevalence of bacterial vaginosis and vaginal flora changes in peri- and postmenopausal women. J. Clin. Microbiol..

[116] Dennerstein G.J., Ellis D.H. (2001). Oestrogen, glycogen and vaginal candidiasis. Aust. N. Z. J. Obstet. Gynaecol..

[117] Gupta S., Kumar N., Singhal N., Kaur R., Manektala U. (2006). Vaginal microflora in postmenopausal women on hormone replacement therapy. Indian J. Pathol. Microbiol..

[118] Fischer G., Bradford J. (2011). Vulvovaginal candidiasis in postmenopausal women: The role of hormone replacement therapy. J. Low. Genit. Tract. Dis..

[119] Tarry W., Fisher M., Shen S., Mawhinney M. (2005). Candida albicans: The estrogen target for vaginal colonization. J. Surg. Res..

[120] Bodnar L.M., Krohn M.A., Simhan H.N. (2009). Maternal vitamin D deficiency is associated with bacterial vaginosis in the first trimester of pregnancy. J. Nutr..

[121] Kaur H., Bala R., Nagpal M. (2017). Role of Vitamin D in urogenital health of geriatric participants. J. Midlife Health.

[122] Ginkel P.D., Soper D.E., Bump R.C., Dalton H.P. (1993). Vaginal flora in postmenopausal women: The effect of estrogen replacement. Infect. Dis. Obstet. Gynecol..

[123] Krysiak R., Gilowska M., Okopien B. (2016). Sexual function and depressive symptoms in young women with low vitamin D status: A pilot study. Eur. J. Obstet. Gynecol. Reprod. Biol..

[124] Inal Z.O., Inal H.A., Gorkem U. (2020). Sexual function and depressive symptoms in primary infertile women with vitamin D deficiency undergoing IVF treatment. Taiwan J. Obstet. Gynecol..

[125] Vitale S.G., Caruso S., Rapisarda A.M.C., Cianci S., Cianci A. (2018). Isoflavones, calcium, vitamin D and inulin improve quality of life, sexual function, body composition and metabolic parameters in menopausal women: Result from a prospective, randomized, placebo-controlled, parallel-group study. Prz. Menopauzalny.

[126] Provvedini D.M., Tsoukas C.D., Deftos L.J., Manolagas S.C. (1983). 1,25-dihydroxyvitamin D3 receptors in human leukocytes. Science.

[127] Adorini L., Giarratana N., Penna G. (2004). Pharmacological induction of tolerogenic dendritic cells and regulatory T cells. Semin. Immunol..

[128] Wang T.T., Nestel F.P., Bourdeau V., Nagai Y., Wang Q., Liao J., Tavera-Mendoza L., Lin R., Hanrahan J.W., Mader S. (2004). Cutting edge: 1,25-dihydroxyvitamin D3 is a direct inducer of antimicrobial peptide gene expression. J. Immunol..

[129] Tian H., Miao J., Zhang F., Xiong F., Zhu F., Li J., Wang X., Chen S., Chen J., Huang N. (2018). Non-histone nuclear protein HMGN2 differently regulates the urothelium barrier function by altering expression of antimicrobial peptides and tight junction protein genes in UPEC J96-infected bladder epithelial cell monolayer. Acta Biochim. Pol..

[130] Sayeed I., Turan N., Stein D.G., Wali B. (2019). Vitamin D deficiency increases blood-brain barrier dysfunction after ischemic stroke in male rats. Exp. Neurol..

[131] Won S., Sayeed I., Peterson B.L., Wali B., Kahn J.S., Stein D.G. (2015). Vitamin D prevents hypoxia/reoxygenation-induced blood-brain barrier disruption via vitamin D receptor-mediated NF-kB signaling pathways. PLoS ONE.

[132] Alperin M., Burnett L., Lukacz E., Brubaker L. (2019). The mysteries of menopause and urogynecologic health: Clinical and scientific gaps. Menopause.

[133] Zhang Q., Zhang Z., He X., Liu Z., Shen L., Long C., Wei G., Liu X., Guo C. (2023). Vitamin D levels and the risk of overactive bladder: A systematic review and meta-analysis. Nutr. Rev..

[134] Schulte-Uebbing C., Schlett S., Craiut D., Bumbu G. (2016). Stage I and II Stress Incontinence (SIC): High dosed vitamin D may improve effects of local estriol. Dermato-Endocrinology.

[135] Dallosso H.M., McGrother C.W., Matthews R.J., Donaldson M.M., Leicestershire M.R.C.I.S.G. (2004). Nutrient composition of the diet and the development of overactive bladder: A longitudinal study in women. Neurourol. Urodyn..

[136] Arjmand M., Abbasi H., Behforouz A. (2023). The effect of vitamin D on urgent urinary incontinence in postmenopausal women. Int. Urogynecol. J..

[137] Brannon-Peppas L. (1993). Novel vaginal drug release applications. Adv. Drug Deliv. Rev..

[138] Van der Straten A., Stadler J., Montgomery E., Hartmann M., Magazi B., Mathebula F., Schwartz K., Laborde N., Soto-Torres L. (2014). Women’s experiences with oral and vaginal pre-exposure prophylaxis: The VOICE-C qualitative study in Johannesburg, South Africa. PLoS ONE.

[139] Garg S., Tambwekar K.R., Vermani K., Kandarapu R., Garg A., Waller D.P., Zaneveld L.J. (2003). Development pharmaceutics of microbicide formulations. Part II: Formulation, evaluation, and challenges. AIDS Patient Care STDS.

[140] Hull T., Hilber A.M., Chersich M.F., Bagnol B., Prohmmo A., Smit J.A., Widyantoro N., Utomo I.D., Francois I., Tumwesigye N.M. (2011). Prevalence, motivations, and adverse effects of vaginal practices in Africa and Asia: Findings from a multicountry household survey. J. Womens Health.

